# Performance of Pyridylthiourea‐Polyethylenimine Polyplex for siRNA‐Mediated Liver Cancer Therapy in Cell Monolayer, Spheroid, and Tumor Xenograft Models

**DOI:** 10.1002/gch2.201700013

**Published:** 2017-05-19

**Authors:** Jean Baptiste Gossart, Etienne Pascal, Florent Meyer, Emilie Heuillard, Mathieu Gonçalves, Francine Gossé, Eric Robinet, Benoît Frisch, Cendrine Seguin, Guy Zuber

**Affiliations:** ^1^ Université de Strasbourg‐CNRS CAMB UMR 7199 Faculté de Pharmacie 74 route du Rhin 67400 Illkirch France; ^2^ Université de Strasbourg‐INSERM UMRS 1121 Biomaterials and Bioengineering, FTMS 11 rue Humann 67000 Strasbourg France; ^3^ Institut Hospitalo‐Universitaire de Strasbourg 1 place de l'Hôpital 67000 Strasbourg France; ^4^ Inserm U1110 3 rue Koeberlé 67000 Strasbourg France; ^5^ Université de Strasbourg‐CNRS, UMR 7242 Boulevard Sebastien Brant 67400 Illkirch France

**Keywords:** delivery system, liver cancer, polo‐like kinase, polyethylenimine, siRNA

## Abstract

Medical application of siRNAs relies on methods for delivering nucleic acids into the cytosol. Synthetic carriers, which assemble with nucleic acids into delivery systems, show promises for cancer therapy but efficiency remains to be improved. In here, the effectiveness of pyridylthiourea‐polyethylenimine (πPEI), a siRNA carrier that favors both polyplex disassembly and endosome rupture upon sensing the acidic endosomal environment, in 3 experimental models of hepatocellular cancer is tested. The πPEI‐assisted delivery of a siRNA targeting the polo‐like kinase 1 into Huh‐7 monolayer produces a 90% cell death via a demonstrated RNA interference mechanism. Incubation of polyplex with Huh‐7 spheroids leads to siRNA delivery into the superficial first cell layer and a 60% reduction in spheroid growth compared to untreated controls. Administration of polyplexes into mice bearing subcutaneous implanted Huh‐7Luc tumors results in a reduced tumor progression, similar to the one observed in the spheroid model. Altogether, these results support the in vivo use of synthetic and dedicated polymers for increasing siRNA‐mediated gene knockdown, and their clinical promise in cancer therapeutics.

## 1 Introduction

Synthetic interfering RNAs (siRNAs) are double‐stranded RNA duplexes able to suppress expression of a gene through a sequence‐selective and enzymatic‐mediated mRNA degradation mechanism occurring in the cytosol of all mammalian cells.^1^ This gene silencing method is mediated by binding of siRNAs to the RNA‐induced silencing complex (RISC)^2^ and can be employed therapeutically in diseases with an identified genetic target. The pharmacological efficacy of siRNA is quite poor for two reasons. First, siRNA does not freely diffuse across the plasma membrane to reach its molecular targets. Second, siRNA is a biodegradable molecule and it is rapidly eliminated by glomerular filtration. Various actions have been undertaken to enhance the pharmacological profile of siRNA. Optimization of the chemical structure of siRNA enhances resistance to nuclease and conjugation to ligands enables hepatocyte targeting.^3^ Aside, the siRNA can be assembled with a carrier into a delivery system. Encapsulating the siRNA with a carrier into a particle improves siRNA stability^4^ and shielding the particle's surface with polyethyleneglycol^5^ and/or tissue‐targeting ligands is an effective mean to improve biodistribution.^6^ Modifying the chemical structure of the carrier can improve the siRNA transfer into the cytosol across the plasma membrane and molecules such as cationic lipids,^7^ polymers,^8^ or lipid‐like molecules^9^ have been made for that purpose. However, properties increasing siRNA resilience in the blood do not typically allow for cytosolic release. Conception of in vivo delivery system is thus challenging. Several delivery systems have nonetheless demonstrated the potential of the siRNA‐mediated gene silencing technology in animal models of disease.^10^ Optimized siRNA formulations were even tested in a phase I clinical trial for treatment of patients with advanced cancers.^11^ The formulations were generally well‐tolerated and provided therapeutic benefits to some patients. However, further improvements are needed.^12^

To improve the siRNA translocation across the plasma membrane, we have used modified polyethylenimine (PEI). PEI is a nucleic acid delivery polymer known for its ability to become protonated in the endosome and for its endosomolytic activity.^13^ The PEI has also been tested in clinical trials.^14^ We noticed that electrostatic association between the siRNA and the water‐soluble PEI was insufficient for maintaining the siRNA/PEI assembly cohesiveness in serum‐containing cell culture medium. Subsequent polyplex anchorage to cell membrane and internalization in endosome in enough quantities is hence compromised. We therefore enhanced the polyplex stability using complementary hydrophobic polymer–polymer interactions by rendering the 25 kDa PEI insoluble at pH > 7.0.^15^ The PEI hydrophobicity was carefully adjusted for triggering solubilization through protonation of the hydrophobic PEI only inside the endosomes. A pH‐controlled intracellular siRNA release is hence obtained. Among several hydrophobic PEIs, we selected pyridylthiourea‐grafted polyethylenimine (πPEI) as our leading pH‐responsive carrier. This modification improves considerably siRNA delivery efficiency in monolayer cell cultures.^16^ In addition, πPEI assembles siRNA into polyplexes with mean diameters slightly below 100 nm, regardless of the πPEI ethylenimine (N) to siRNA phosphate (P) N/P ratio^16^ and showed promises for in vivo siRNA delivery.^17^ However, The πPEI has not been tested for a siRNA‐mediated tumor therapeutic experiment.

Hepatocellular cancer (HCC) represents a major health concern. In 2012, the World Health Organization estimated HCC 745 000 deaths worldwide, encompassing ≈9.1% of all cancer deaths.^18^ Recent reports in the United States also establish hepatic cancer as a major burden. From 2003 to 2012 liver cancer‐associated death rates increased, compared to a reduced overall cancers morbidity.^19^ HCC is frequently associated with cirrhosis, which can make the liver extremely sensitive to hazardous substances. SiRNA delivery systems preferentially accumulate in the liver (either in hepatocytes or Kupffer's cells) upon intravenous administration.^20^ This tropism might raise concerns of adverse liver damage even if the tropism for liver may be reduced by equipping the nanoparticles with a tumor‐targeting element.[[qv: 6b]] An alternative is to administer the nanoparticles into the tumor by performing weakly invasive surgical procedures.^21^ Before engaging in such surgical procedure, our objective was to provide evidence of an antitumoral activity of siRNA/πPEI polyplexes in hepatocellular carcinoma models using a local administration procedure. The polo‐like kinase 1 was chosen because this protein is essential during cell division and is a feasible target for cancer therapy.[[qv: 10c,22]] The siRNA/πPEI polyplexes were evaluated in two in vitro HCC models and in one in vivo model to identify translational performance. The first model was the classical in vitro 2D culture of Huh‐7 cells onto plastic substrates. The second model was Huh‐7 spheroid culture, in which in their 3D growth displays aspects like endogenous tumor morphology.^23^ The third model consisted of subcutaneously implanted Huh‐7 tumor mouse.^24^ Our results confirmed the siRNA delivery efficiency of πPEI‐based systems both in vitro and for in vivo following topical administration. Moreover, the Huh‐7 spheroid cultures appeared more predictive of in vivo performance at least in terms of efficiency. The spheroid in vitro model may be helpful to choose more effective carriers.

## 2 Results and Discussion

### 2.1 Description of the Experimental Setting

**Figure 1:**
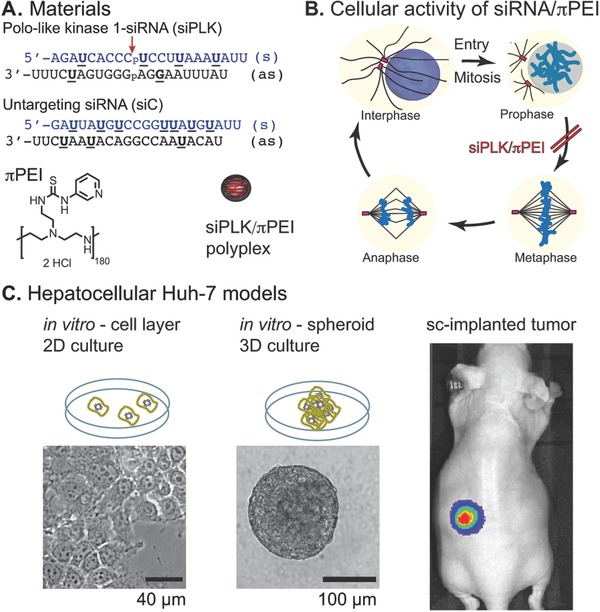
Illustration of described experiments. A) Sequence of the polo‐like kinase 1 (PLK‐1) and control siRNAs. The underlined nucleotides are 2′OMe. The arrow indicates the cleavage site on the targeted PLK‐1 mRNA. B) Representation of the mitotic cell cycle. Inhibition of the PLK‐1 with siRNA blocks the cells entering mitosis in prophase. C) Illustration of the different hepatocellular cancer (HCC) models that were used.

The antitumoral activity of siRNA in HCC has been evaluated using various genetic targets and delivery systems. Kawata et al. demonstrated that a polyplex of atelocollagen with siRNA targeting the polo‐like kinase 1 (siPLK) reduced mouse liver metastatic proliferation.^25^ Judge et al. describes the antitumoral efficacy of stable nucleic acid particles containing a chemically modified siPLK for treatment of mouse hepatic tumor models.[[qv: 10c]] Li et al. evaluated several lipidic formulations and genetic targets and suggested that one novel lipid formulation and a siRNA targeting CDCA1 are efficient for HCC treatment.^26^ Tabernero et al. reported an encouraging outcome from a phase I clinical trial describing both safety and efficacy of ALN‐VSP, a stable lipid formulation containing two siRNAs that target the vascular endothelial growth factor and the kinesin spindle protein.^11^ We decided to target the polo‐like kinase 1 for the following four reasons. First, the polo‐like kinase 1 (PLK‐1) plays critical role during mitotic progression.^27^ Its inhibition blocks cell cycle in mitosis and may induce apoptosis.^28^ Second, PLK‐1 elevations are observed in many tumor‐types, where it is predictive of a poor prognosis.^29^ Third, several investigations at preclinical stage^30^ including two on HCC models,[[qv: 10c,24a]] confirmed the antitumoral potential of targeting PLK1 using the siRNA technology. Fourth, optimized siRNA sequences (including the control) and 2′OMe nucleotides chemical modification that minimize immune response were described (**Figure** **1**A).[[qv: 10c]] At the cellular level, siRNA‐mediated polo‐like kinase 1 inhibition blocks the cell mitosis as early as the prophase (Figure 1B) and causes a typical nuclear morphology that is easy to observe using microscope. The human hepatoma cell line Huh‐7 can be grown in either 2D and 3D cultures, and implanted into immunodeficient mice (Figure 1C). Implanted Huh‐7 tumors can also constitutively express a firefly luciferase for a longitudinal detection of tumor growth using a luminescence in vivo imaging system.[[qv: 24b]] Regarding the delivery system, the siRNA/πPEI polyplexes were always assembled in 4.5% glucose, pH 6.3 using concentrated 0.2 m πPEI. This condition produces an homogenous colloidal suspension of spherical particles with mean diameters slightly below 100 nm, regardless of the πPEI ethylenimine (N) to siRNA phosphate (P) N/P ratio.^16^

### 2.2 Gene Silencing into 2D Culture Model

We initially tested the ability of the siPLK and πPEI to selectively silence the polo‐like kinase 1 in the Huh‐7 hepatoma cells cultivated in the 2D classical setting. Under this experimental condition, adherent cells have a large portion of the outer plasma membrane surface exposed toward the medium, allowing direct and unrestricted cellular anchorage of particles. The polyplexes containing either the control siRNA (siC) or the siPLK were assembled in 4.5% glucose at a N/P ratio of 125. The polyplexes were then directly added to the cells by dilution with the serum‐containing cell culture medium. Total mRNA was then extracted at various time points after polyplex addition. PLK‐1 mRNA levels were quantified by real‐time polymerase chain reaction (PCR). Normalization of the PLK‐1 mRNA levels was performed versus the GAPDH, PSMB2, and SNRPD3 housekeeping genes.^31^

**Figure 2:**
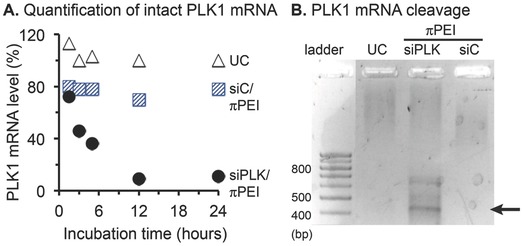
Demonstration of the efficiency of siRNA‐mediated gene silencing activity using siRNA/πPEI polyplexes. A) Quantification of intact PLK1 mRNA level in untreated Huh‐7 cells (UC, white triangles), siC/πPEI (blue hatched squares) or siPLK/πPEI (black dots)‐treated cells. The siC and siPLK correspond to untargeted and polo‐like kinase 1‐targeting siRNAs. Final concentrations were at 20 × 10^−9^ m siRNA and 100 × 10^−6^ m πPEI. Complexes were added directly to Huh‐7 cell monolayer in serum containing cell culture medium. B) 5′RACE‐PCR analysis of polyplexes‐treated cells to detect of the siPLK/RISC‐promoted mRNA fragmentation. The arrow points to the expected length of the siPLK‐specific RACE‐PCR product.

**Figure 3:**
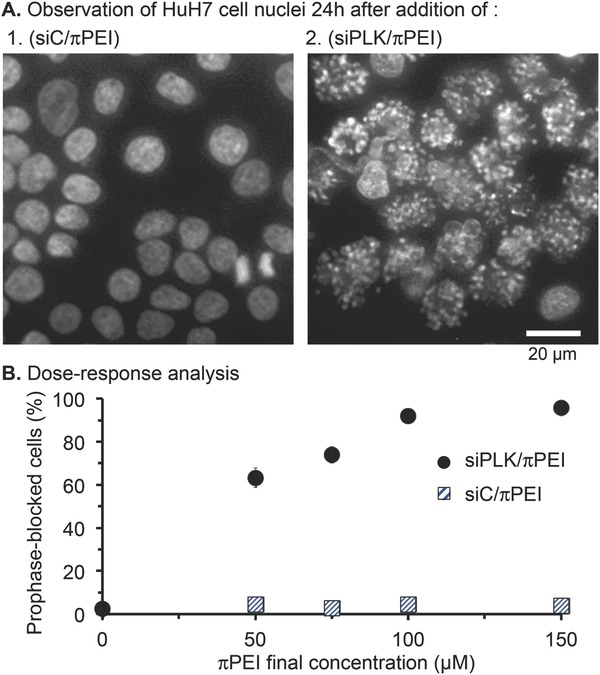
Cellular response to siRNA‐mediated PLK1 mRNA degradation 2 d after addition of siRNA/πPEI onto Huh‐7 2D cultures. A) The morphology of the cell nuclei was observed after staining with Hoechst 33342. Final concentrations were at 20 × 10^−9^ m siRNA and 100 × 10^−6^ m πPEI. B) Analysis of prophase‐blocked Huh‐7 as a function of πPEI concentration. The siRNA concentrations were fixed at 20 × 10^−9^ m and the polyplexes were added 48 h before analysis.

The PLK‐1 mRNA expression was reported as a percentage versus untreated cells (**Figure** **2**A). In the untreated and siC/πPEI‐treated samples, the PLK1 mRNA levels remained roughly constant at all time points. In the siPLK/πPEI‐treated samples, the level of intact PLK1 mRNA rapidly decreased. A 50% diminution was already obtained 3 h after polyplex addition and the maximum 90% diminution was reached in 12 h. During RNA interference the siPLK typically acts in the RNA‐induced silencing complex (RISC) to promote a sequence‐selective cleavage of mRNA targets between the nucleotides 9 and 10 from the siRNA sense sequence (arrow, Figure 1A). To confirm this mechanism of RNA interference, we isolated the 5′phosphate‐RNA fragment using the 5′‐RACE‐PCR assay.[[qv: 10c]] Here, the size of the 5′‐RACE‐PCR product corresponding to the siPLK‐cleaved mRNA fragment was predicted to be 441 bp. The gel electrophoresis analysis showed that only the siPLK/πPEI‐treated Huh‐7 cells produced DNA fragments amplification (Figure 2B). The size of the major fragment corresponded to its predicted size, providing further evidence that the diminished PLK1 mRNA level was caused by a RNA interference mechanism. Next, the therapeutic activity of siPLK in 2D culture was assayed by staining the cellular DNA with Hoechst 33342 2 d after addition of siRNA/πPEI polyplexes (siRNA being either siC or siPLK) (**Figure** **3**A). The cell nuclei in the siC/πPEI‐treated experiments looked similar in both shape and proportion of mitotic cells to untreated Huh‐7 cells (Figure 3A1). πPEI‐delivered siPLK induced dramatic nuclear modifications in over 90% of cells. Genomic DNA appeared fragmented, indicating an irreversible block in mitotic progression as early as prophase, leading to apoptosis as indicated by substantial DNA fragmentation during longer incubation times (see Figure S1 in the Supporting Information). We performed next a dose–response experiment variation of πPEI concentration and maintained the concentration of siRNA at 20 × 10^−9^ m (Figure 3B). The percentage of mitosis‐arrested cells increased with increasing πPEI concentration and reached a maximum of an over 90% mitotic blockage at 100 × 10^−6^ m πPEI.

Overall, these experiments confirmed that πPEI is able to deliver siPLK into Huh‐7 cells with excellent efficiency and leads to a mitotic blockage via a siPLK1/RISC‐induced mRNA fragmentation.

### 2.3 Gene Silencing in 3D Cultured Cells (Spheroids)

**Figure 4:**
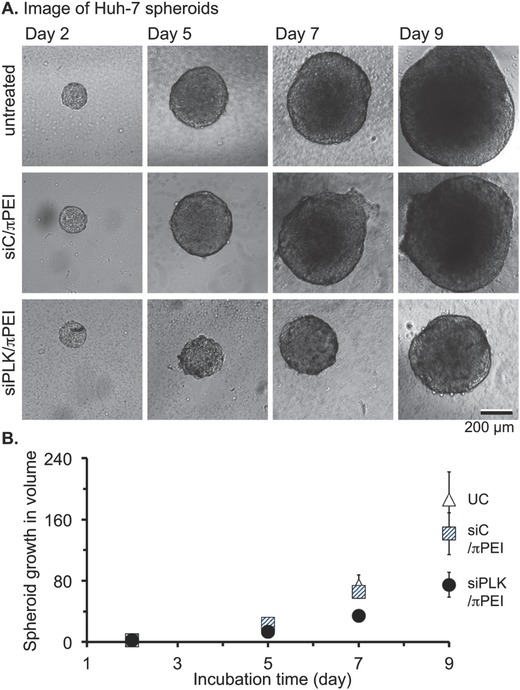
In vitro growth of Huh‐7 3D tumors in presence of siRNA polyplexes. A) Representative images of spheroid's growth over time in absence or presence of siC/πPEI and siPLK/πPEI. B) Plot showing the average (± SD, *n* = 7) volumetric growth over time of untreated spheroids (white triangles) or ones that were incubated with siC/πPEI (blue hatched squares) or siPLK/πPEI (black dots). The spheroid volume (%) was reported relative to the first day.

Since the siPLK/πPEI polyplex can efficiently block Huh‐7 proliferation, we next evaluated the performance and behavior of the polyplexes for siRNA delivery in 3D tissue culture. Tumor spheroids consist of multiple cells grown into a sphere and are in vitro model for tumorogenesis especially at early states.^32^ Huh‐7 spheroids were grown using a hanging drop method^33^ until diameters reach ≈120–150 μm. These spheroids were then incubated with siRNA/πPEI polyplexes and their growth over 9 d was determined by measuring spheroid volume from calibrated images (**Figure** **4**). Between the days 2 and 9, untreated spheroid volume increased roughly 20 folds. The volumes of siC/πPEI‐ and siPLK/πPEI‐treated spheroids showed 20% and 60% growth reductions, respectively.

**Figure 5:**
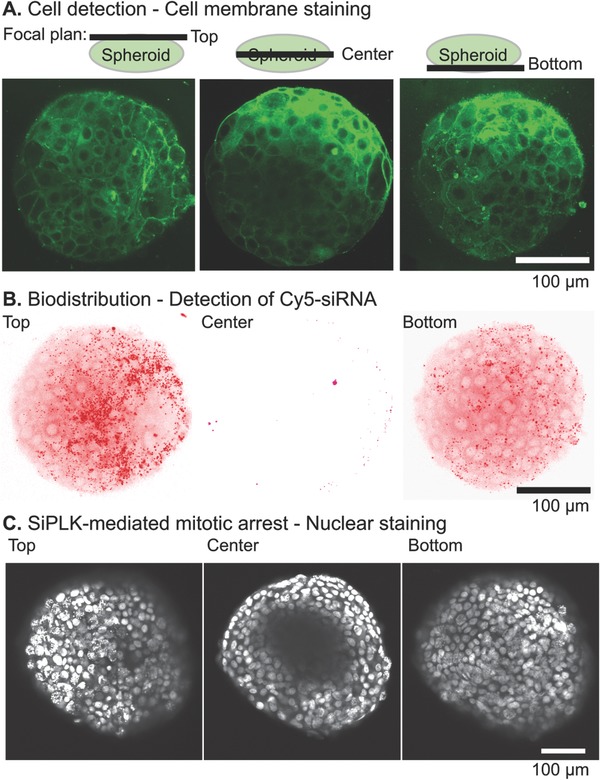
Visualization of siRNA/πPEI delivery within the spheroid. A,B) Rhodamine‐siRNA/πPEI polyplexes (N/P 14) were incubated 24 h with a spheroid in the cell culture medium containing serum at final concentrations of 180 × 10^−9^ m siRNA and 100 × 10^−6^ m πPEI. The spheroid was then fixed with paraformaldehyde and the cell membranes were stained with PKH67 fluorescent cell linker kit. The spheroid was observed at three focal plans for visualization of the cell membrane (A, green fluorescence) or for visualization of the Cy5‐siRNAs polyplexes (B, red fluorescence). C) The Huh‐7 spheroid was incubated with siPLK/πPEI for 9 d. The spheroids were fixed and the nuclei stained with Hoechst 33342.

The localization of the siRNA polyplexes within the spheroids was assayed by incubating Huh‐7 spheroids with a πPEI polyplex containing a Rhodamine‐labeled siRNA during 24 h. After membrane staining with pKH67, confocal imaging was performed (**Figure** **5**A,B). Fluorescently labeled siRNA/πPEI polyplexes remained bound to the spheroid surface, but do not penetrate inside the sphere core. Incubation of the spheroid with siPLK1/πPEI polyplexes produced a similar result (Figure 5C). Nuclear fragmentation typical of polo‐like kinase 1 gene silencing was observed only in the superficial cell layers. These results are in accordance with previous works showing that polyplex^34^ and, more generally particles sized over 5 nm, do not easily reach the interior of the tumor.^35^ Altogether, these data demonstrate that the polyplexes can only bind and deliver siRNA to the first outer cell layer of the spheroid. Reduced internal accessibility maybe why siPLK polyplexes reduce spheroid proliferation but do not induce full regression.

### 2.4 In Vivo Evaluation of πPEI‐Delivered siRNA

**Figure 6:**
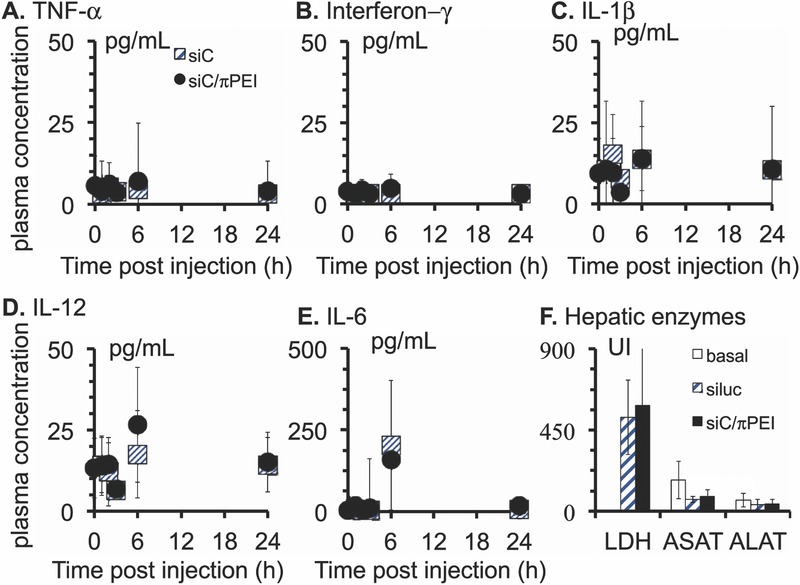
In vivo evaluation of the immune response to injected polyplexes. Immunocompetent Balb/c mice were intravenously injected with 1 mg kg^−1^ siC (hatched blue square) or siC/πPEI (black dots). A–E) Blood samples were collected at various times postinjection and plasma concentrations (in pg mL^−1^) were determined for TNF‐α, interferon γ, IL‐1β, IL‐12, and IL‐6. F) The presence of hepatic enzymes in the plasma was measured 24 h after injection. Data are expressed as mean ± SD (*n* = 8). The basal plasma levels (white bar) of each component were measured in blood samples collected 48 h before injection. For convenience, this value was plotted at 0 h.

Nanoparticular delivery systems can elicit production of proinflammatory cytokines (TNF‐α, IFN‐γ, and IL‐12) and a cascade of immune response events causing toxicity but also, in some cases, tumor growth reductions.^36^ We evaluated the potential of siRNA/πPEI polyplexes to trigger an inflammatory response in healthy immunocompetent Balb/c mice by measuring release of several proinflammatory cytokines. The 2′‐OMe‐modified SiC was reported not to trigger release of proinflammatory cytokines in animal.[[qv: 10c]] It was hence associated with πPEI. The polyplex (N/P 14, 20 μg siRNA; 70 μg πPEI/mouse) was intravenously injected into the tail vein of Balb/c mice and blood samples were collected at different time points for quantification of plasma cytokine concentrations (**Figure** **6**). Administration of the siC alone, or in complex, did not trigger release of TNF‐α, IFN‐γ, IL‐1β, and IL‐12 within a 24 h period. A transient plasma release of IL‐6 was observed at 6 h when the siRNA was provided alone or in complex with πPEI. This induction (160 pg mL^−1^) was moderate compared to lipopolysaccharide (>24 000 pg mL^−1^) or siRNA/PEI polyplexes (>1200 pg mL^−1^) response.^37^ The fate of intravenously injected Cy5‐siRNA/πPEI polyplexes was longitudinally monitored using a fluorescence in vivo imaging system (Figure S2, Supporting Information). Polyplexes accumulated in the liver as expected for nanoparticular systems.^38^ Hepatic damage was evaluated by measuring the plasma levels of alanine aminotransferase (ALAT), aspartate aminotransferase (ASAT), and cytosolic lactate dehydrogenase (LDH) 24 after injection (Figure 6A). Plasma ALAT and ASAT levels were low, indicating siC/πPEI does not promote acute liver damage at tested dosages. A LDH activity (500 U L^−1^) was detected in the plasma but was comparable to the response triggered by injection of PEI.

**Figure 7:**
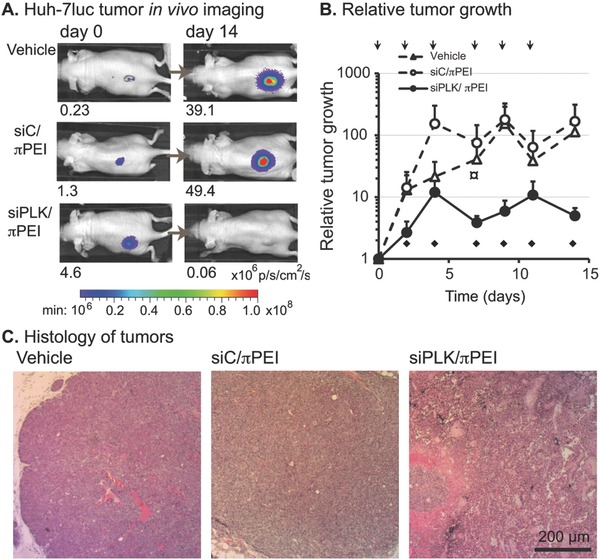
Evaluation of antitumoral activity of siPLK/πPEI in a mice model of hepatocellular cancer. A) In vivo bioluminescence imaging of representative mice implanted with subcutaneous Huh‐7‐Luc tumors. The bioluminescence activity, expressed as p s^−1^ cm^−2^ sr^−1^, is indicated under each image. B) Quantification of relative tumor growth. The vehicle alone (glucose solution), siC/πPEI, and siPLK/πPEI (20 μg siRNA; 70 μg πPEI) were repeatedly injected into tumors over two weeks at times indicated by the arrows. The relative tumor growth (RTG) was calculated as the ratio of luciferase activity at d14 to the luciferase activity at d0. Bioluminescence imaging acquisitions were performed before products' injections. The mean ± SE luciferase activity at day 0 for the vehicle, siC/πPEI, and siPLK/πPEI groups was, respectively, 3.8 × 10^6^, 4.6 × 10^6^, and 6.5 × 10^6^ p^−1^ s^−1^ cm^−2^ sr^−1^ and RTG is expressed as mean ± SE of ratios between luciferase activity at the indicated day to the luciferase activity at d0 (*n* = 12/group). The diamond (♦) indicates significant variation between groups (*p* < 0.05) using a Mann–Whitney test. C) Hematoxylin/eosin staining of tumors at day 14 after the indicated treatment.

We next evaluated siPLK/πPEI polyplexes performance in a hepatic cancer model where immunodeficient nude mice were engrafted with luciferase‐expressing Huh‐7 tumors.[[qv: 24b]] In an initial experiment, luciferase‐expressing Huh‐7 xenografts were established orthotopically near the liver of immunodeficient mice. The siRNA/πPEI polyplexes (N/P 14) containing a Cy5‐siRNA were then intravenously injected. Differential accumulation of the polyplex into the tumor and in the liver was measured 24 h after injection (Figure S3, Supporting Information). The polyplex was detected in both the liver and the nearby tumor. Polyplex tumor accumulation was moderate and heterogeneous, confirming that the iv administration of cationic siRNA/πPEI polyplex is not effective. We then evaluated the performance of siRNA/πPEI polyplex injected directly into the tumor of subcutaneous luciferase‐expressing Huh‐7 xenograft mouse tumors. Three weeks after implantation, the tumor bioluminescence was measured in 3 experimental groups: siPLK/πPEI, siC/πPEI, and vehicle (4.5% glucose solution). All groups followed a treatment protocol consisting of 6 intratumoral administrations at days 0, 2, 4, 7, 9, and 11 of either the vehicle alone (4.5% glucose solution, 50 μL), siC/πPEI or siPLK/πPEI polyplexes (20 μg siRNA, 50 μg πPEI, 50 μL). During this time, the tumor growth was evaluated by measuring the bioluminescence of Huh‐7‐Luc tumors in anesthetized mice (**Figure** **7**A). This treatment regime did not induce weight loss, nor adverse behavioral modifications. Both at the beginning and end of treatments protocol siPLK/πPEI‐treated mice showed a low luminescence signals emitted by the siPLK/πPEI‐treated tumors. After normalization of the bioluminescence level, the relative tumor growth (RTG) of each group was plotted over the treatment time course (Figure 7B). SiPLK/πPEI significantly diminished the tumor growth from 7 d onward versus control groups (*p* < 0.05, Mann–Whitney rank sum test). Using the same administration protocol, controls were performed using siRNA/PEI polyplexes containing unmodified PEI or sticky siRNA^39^ (Figure S4, Supporting Information). With these complexes, diminished tumor growths were also obtained, but were not specific to the polo‐like kinase 1 siRNA. At the end of the treatment protocol (day 14), the mice were euthanized. The tumors were excised and were subjected to histological analyses (Figure 7C).

Necrosis was blindly scored and higher levels were observed in the PLK1/πPEI‐treated tumors compared with controls. The PLK1mRNA levels within treated tumors were finally quantified using RT‐PCR after a single injection of the polyplexes containing siC or siPLK (Figure S5, Supporting Information). A 40% reduced PLK1 mRNA level was observed only in the siPLK/πPEI‐treated group, suggesting that the antitumoral activity is mostly promoted by siPLK1 delivery with πPEI.

## 3 Conclusion

The usefulness of polyethylenimine as a carrier for nucleic acids has been evaluated in phase I/II clinical trials in patients with invasive bladder tumors[[qv: 14a]] or advanced pancreatic cancer.[[qv: 14b]] In both trials, nucleic acid/PEI polyplexes were administrated locally. No serious adverse effects were observed indicating that local administration coupled to a surgical procedure may provide benefits. We showed here that pyridylthiourea‐polyethylenimine assists the antimitotic activity of a siRNA targeting the polo‐like kinase 1 into the Huh‐7 cell line via an RNA interference mechanism. πPEI delivery efficiency was maximal when the polyplexes were incubated onto cell grown in vitro as monolayer. Here over 90% RNAi‐mediated cell‐arrest and cell death was obtained. When the polyplexes were incubated with Huh‐7 grown as spheroid, delivery performance was reduced by about 60% likely as only the superficial cells of the spheroid were accessible. Intratumoral administration of the polyplexes into implanted hepatic tumors slowed tumor growth, an antitumoral effect linked to diminished intracellular polo‐like kinase 1 mRNA levels. Administration of polyplex into fully immunocompetent Balb/c mice triggered a minimal release of proinflammatory cytokines. Altogether, these results support the medical application of nucleic acid delivery systems based on polyethylenimine when topical administration is feasible. We believe that the progress in surgery procedures renders topical applications more and more feasible and should open opportunities for blood‐incompatible but efficient in vitro siRNA delivery systems. Our results also clearly indicate that polyplex does not reach buried cell surface on their own but likely relies on cellular mobility or a tumor invasion of the injected sites for efficacy.

## 4 Experimental Section

Detailed experimental procedures are reported in the Supporting Information. Animal experimentations were performed in accordance with European recommendations (Directive 2010/63/UE, September 22, 2010) and French regulations (Décret 2013‐118, February 1, 2013). They received the approval no. 00465.02 from the French Ministry of Higher Education and Research in date of March 11, 2014.

## Conflict of Interest

The authors declare no conflict of interest.

## Supporting information

Supplementary
