# Supplementary material for: Performance of Pyridylthiourea‐Polyethylenimine Polyplex for siRNA‐Mediated Liver Cancer Therapy in Cell Monolayer, Spheroid, and Tumor Xenograft Models
Source: Glob Chall. 2017 May 19;1(4):1700013. doi: 10.1002/gch2.201700013 (PMC6607116; doi:10.1002/gch2.201700013)
Supplement: Supplementary file 1 — Supplementary [file GCH2-1-1700013-s001.pdf]

Copyright WILEY-VCH Verlag GmbH & Co. KGaA, 69469 Weinheim, Germany, 2017.

Global Challenges
Open Access

Supporting Information

for *Global Challenges,* DOI: 10.1002/gch2.201700013

Performance of Pyridylthiourea-Polyethylenimine Polyplex for siRNA-Mediated Liver Cancer Therapy in Cell Monolayer, Spheroid, and Tumor Xenograft Models

*Jean Baptiste Gossart, Etienne Pascal, Florent Meyer, Emilie Heuillard, Mathieu Gonçalves, Francine Gossé, Eric Robinet, Benoît Frisch, Cendrine Seguin, and Guy Zuber**

Copyright WILEY-VCH Verlag GmbH & Co. KGaA, 69469 Weinheim, Germany, 2016.

Supporting Information

Performance of Pyridylthiourea-Polyethylenimine Polyplex for siRNA-Mediated Liver Cancer Therapy in Cell Monolayer, Spheroid and Tumor Xenograft Models

Jean Baptiste Gossart, Etienne Pascal, Florent Meyer, Emilie Heuillard, Mathieu Gonçalves, Francine Gossé, Eric Robinet, Benoît Frisch, Cendrine Seguin, Guy Zuber*

1. Experimental procedures

1.1. General

Dynamic Light Scattering (DLS) measurements were performed using a NanoZS apparatus (Malvern Instruments, Paris, France) at 25°C. The pH of solutions was measured with a Radiometer pHmeter PHM240 (Radiometer Analytical, Villeurbanne, France) and an InLab Micro electrode (Mettler Toledo, Viroflay, France). Fluorescence observations were carried out with a Zeiss LSM 510 microscope using a 40 X Zeiss Achroplan objective.

Plastics tubes were used as purchased and guaranteed sterile and RNAse-free. RNAse-free water was purchased from Millipore (Molsheim, France). Branched PEI 25 kDa (reference 40, 872-7, batch 09529KD-466) and other chemicals unless specified were purchased from Sigma-Aldrich (St Quentin, France). The pyridylthiourea-grafted PEI (πPEI) was prepared by reacting 30% of the ethylenimine residues of 25 kDa PEI with 3-pyridylisothiocyanate and as hydrochloride salt after dialysis and lyophilisation [1]. Before experiments, the polymer hydrochloride salt was dissolved in RNAse-free water and the pH was adjusted to 6.3 with NaOH 1M. The stock solution was set at 200 and 20 mM in ethylenimine residues using an absorption coefficient of 2660 $M^{-1}.cm^{-1}$ at 245 nm. Experiments involving cell lines as well as preparation of solutions were performed under biosafety level-2 conditions. The human hepatocellular Huh7 cell line was maintained at 37 °C in a 5 % CO_2 humidified atmosphere.

The cell line was grown in an adherent state onto a plastic substrate (175 cm^2 Falcon tissue culture flask) in high-glucose Dubelcco’s modified eagle medium (DMEM) supplemented with 10 % fetal bovine serum (FBS) (Perbio, Brebières, France), 1 % Non-essential amino acids, 50 U/mL penicillin G and 50 µg/mL streptomycin. When needed, cells were suspended in solution by a trypsin treatment. The siRNA were purchased from Eurogentec (Seraing, Belgique), annealed at 90 µM (or 1.23 µg.$µL^{-1}$) and stored in aliquots at -80°C. The sense (S) and antisense sequences (AS) of the siRNA duplexes were as followed. Untargeted siRNA (siC): 5’-GAUU AUGU CCGG UUAU GUAU U (S) and 5’-UACA UAAC CGGA CAUA AUCU U (AS). The siC sequences are the ones of Luc-U that was described by Judge et al. [2]. Important to mention, this 2’OMe modified siRNA did not target and silence the luciferase gene of Huh-7luc cells. It did not trigger as well an immune response [2]. Polo-like kinase 1 siRNA (siPLK): 5’-AGAU CACC CUCC UUAA AUAU U (S) and 5’-UAUU UAAG GAGG GUGA UCUU U (AS). Underlined nucleotides are 2’OMe nucleotides. Fluorescently labeled siC: 5'-Rhodamine-CUUA CGCU GAGU ACUU CGA-d(TT) (S) and 5’-UCGA AGUA CUCA GCGU AAG-d(TT) (AS).

1.2. *In vitro* siRNA delivery into 2D culture

The Huh-7 cells were seeded the day before the experiments. Transfection assays were performed either in 6-well plate (Costar, 100000 cells/well, 2 mL medium) or in 12-well plate (Costar, 50000 cells/well, 1 mL medium). This procedure is described for assays at final concentrations of 100 µM πPEI and 20 nM siRNA. These concentrations correspond to an ethylenimine Nitrogen (N) to siRNA Phosphate (P) N/P ratio of 125. In experiments using other πPEI concentrations, the πPEI input was scaled accordingly. For polyplex formation, the 100 µM siRNA stock solution was freshly diluted in 4.5% glucose solution to 2.2 µM. This 2.2 µM siRNA solution (20 µL) was then rapidly added to 200 mM πPEI (1.1 µL). After

a gentle vortex, the mixture was diluted with DMEM (180 µL) and immediately added into wells by dilution into the complete cell culture medium. Added volumes were 200 µL for the 6-well plate, 100 µL for the 12-well plate. The living cells were stained with Hoechst 33342 for nuclei imaging by incubation into cell culture medium containing serum for 30 minutes (1 µg/mL final concentration). The cell culture was carefully removed and replaced with PBS for immediate imaging.

1.3. Quantification real time polymerase chain reaction

Experiments were performed in 6-well plates. At the end of the incubation time, the cell culture medium was removed and cells were lysed in 400µL TRI reagent (Sigma aldrich). For *in vivo* experiment, subcutaneous Huh-7-Luc tumors (see below) were collected 3h after a single intratumoral injection of siRNA/πPEI and were lysed in RLT buffer (Qiagen, Courtaboeuf, France) using a MACS Dissociator and M tubes (Miltenyi Biotec, Paris, France). Total RNAs were then isolated with the DirectZol® Kit (Zymoresearch, Irvine, CA). The RNA was quantified using the Nanodrop1000 and aliquots (0.5 µg) were reverse-transcribed using the Maxima First Strand Synthesis Kit (Thermo Fisher Scientific Inc.) and the real time-PCR was performed using the TaqMan® technology and was normalized using GAPDH, PSMB2 and SNRPD3 as housekeeping genes. TaqMan primers were Hs00153444_m1 (PLK1), Hs02758991_g1 (GAPDH), Hs001188207_m1 (SNRPD3), Hs01002946_m1 (PSMB2).

1.4. 5’ RNA ligase-mediated RACE.

The 5’ RNA Ligase-Mediated-Rapid Amplification of cDNA Ends (5’ RLM RACE) was performed according to the published protocol with slight modifications using the GeneRacer kit (Invitrogen). Briefly, cellular RNA isolated as previously described for real-time PCR (17 µg) was mixed with the GeneRacer RNA adaptor (5′-CGAC UGGA GCAC GAGG ACAC

UGAC AUGG ACUG AAGG AGUA GAAA; 250 ng), heated to 65°C for 5 minutes, and snap-cooled on ice prior to ligation. RNA ligation was performed at 37°C for 1 hour in 1× ligase buffer, 30 U RNaseOut (Invitrogen), and 30 U RNA ligase (Ambion Inc.). Samples were then purified by phenol/chloroform extraction and Ethanol precipitation. RNA ligation product (2 µL-aliquot) was reverse transcribed using SuperScript III (Invitrogen) and a PLK1-specific primer (5′-GGAC AAGG CTGT AGAA CCCA CAC) at 55°C for 50 minutes. The enzyme was inactivated at 70°C for 15 minutes and the mixture was snap-cooled on ice. The PCR was performed using forward (GR5: 5′-CGAC TGGA GCAC GAGG ACAC TGA) and reverse (PLK1424rev: 5′-CCAG ATGC AGGT GGGA GTGA GGA) primers on a PikoReal apparatus (Thermo Fisher Scientific Inc). The PCR conditions were: 94°C for 2 minutes (1 cycle), 94°C for 30 seconds and 72°C for 1 minute (5 cycles), 94°C for 30 seconds and 70°C for 1 minute (5 cycles), 94°C for 30 seconds, 65°C for 30 seconds and 68°C for 1 minute (25 cycles), and 68°C for 10 minutes (1 cycle). The PCR products were then analyzed by electrophoresis on a 2% agarose gel (TBE) and visualized after SYBR safe staining (Life Technologies).

1.5. Preparation of Huh-7 tumor spheroid

Prior to the culture, sterile 96-well plates equipped with a lid (Corning™ Costar™ Flat Bottom Cell Culture Plates, Fisher scientific, Ref 10792552) were coated with poly(2-hydroxyethyl methacrylate) (poly-HEMA; sigma P3932-10G). The coating was done by addition of a 0.5 % (w/v) poly-HEMA solution in ethanol (100 µL) into each well of a 96-well plate at 38°C. The plate was kept at 38°C for 24h for complete ethanol evaporation and for ensuring surface coating. PBS (100 µL) was then added into each well. Adherent Huh-7 were treated with trypsin and suspended in complete medium at 5.3 10^3 cell/mL. The cell suspension (15 µL) were deposited on the inside face of the 96-well plate lid. The lid was then replaced on the PBS-filled plate and the cells in the hanging drop were led to grow in the

incubator for 3 days. The PBS was then discarded and replaced with complete cell culture medium (100 µL). Each drop containing one single spheroid was then transferred from the lid into the well by centrifugation at 500 RPM for 1 minute. These tumor spheroids were then immediately used for siRNA delivery experiments or kept in culture for further growth.

1.6. siRNA polyplex formation for tumor delivery

Polyplexes were prepared in 4.5% glucose at a N/P ratio of 14. Briefly, the 90 µM siRNA stock solution (80 µL) was mixed with a 9 % (w/v) glucose solution (100 µL) The mixture was then rapidly added to the 0.2 M πPEI stock solution, pH 6.3 (20 µL). The size and zeta potential of the polyplex after 50 times dilution in 4.5% glucose were determined at 95 nm and +27 mV from DLS data. The polyplex solution (0.5 $\mu g.\mu L^{-1}$ in siRNA, 1.68 $\mu g.\mu L^{-1}$ in πPEI) was further diluted in 4.5% glucose solution to 0.4 $\mu g.\mu L^{-1}$ in siRNA, 1.4 $\mu g.\mu L^{-1}$ in πPEI for intratumor administration.

1.7. siRNA delivery into tumor spheroids

The experiments were carried out in quintuplicate in 96-well plate using the spheroids as previously described. The siRNA polyplexes as previously prepared in 4.5% glucose were diluted 20 times in complete cell culture medium. The polyplexes N/P 14 (11 µL) were then immediately added to each spheroid by further dilution into the cell culture medium (100 µL). Final concentrations were 180 nM siRNA and 100 µM πPEI. The cell culture medium was replaced at days 2, 5 and 7 with complete cell culture medium containing polyplexes.

The spheroid growth was examined using a Nikon Eclipse TS100 microscope and a 10 X objective. Images were recorded at days 0, 2, 5, 7 and 9 with a Nikon Color CCD DS-Fi2 Camera and a DS-L3 camera controller. The area (A) of each spheroid was measured using the NIH Image J software and reported in μm^2. The volume (V) was then calculated using the

formula: $V = 4/3(A)^{3/2}$ The MCTS growth (G) was calculated relative to the initial volume (V0; day 0). An ANOVA statistical analysis followed by a Bonferroni's test was performed between groups at the days 7 and 9. Comparison showed statistic differences ($p < 0.005$) between the siPLK/πPEI group and the controls (siC/πPEI or untreated groups). No statistical variation was seen between the two controls.

1.8. Evaluation of particle diffusion in Huh-7 spheroids

The experiments were carried in triplicate. Huh-7 spheroid was incubated in a complete cell culture medium for 24 h with Rho-SiC/πPEI polyplexes N/P 14 at final concentrations of 180 nM siRNA and 100 µM πPEI. The spheroids were then fixed by addition of 8% (w/v) paraformaldehyde (EMS group, Hatfield, PA, USA) in PBS (100 µL) to each well. After 15 min, fixed Huh7 spheroids were harvested with a 400 µm fiber loop and incubated for 5 min in PBS (100 µL). The cell membranes were then stained using the green fluorescent PKH67GL staining kit (Sigma Aldrich, St Quentin, France) and the described procedure. After membrane staining, the tumors were rinsed in PBS and were mounted in an Antifade Mounting Medium (Vector laboratories, H-1000, Burlingame, CA). For imaging, excitation wavelengths were set at 488 nm and 543 nm for triggering membrane stain and rhodamine fluorescence, respectively. Green and red light emissions were recorded after filtration using 505-530 nm band-pass and 585 nm long pass filters. Several images were recorded along the z-axis for focal sectioning.

1.9. Evaluation of siRNA-mediated PLK1 gene silencing in Huh-7 spheroid:

The experiments were carried in triplicate. Huh-7 spheroid was incubated in a complete cell culture medium for 9 days with siPLK/πPEI polyplexes N/P 14 at final concentrations of 180 nM siRNA and 100 µM πPEI. The cell culture medium was replaced at days 2, 5 and 7 with

complete cell culture medium containing polyplexes. After 9 days, the spheroids were fixed by addition of 8% (w/v) paraformaldehyde (EMS group, Hatfield, PA, USA) in PBS (100 µL) to each well. After 15 min, fixed Huh-7 spheroids were harvested with a 400 µm fiber loop, incubated for 5 min in PBS (100 µL) and the cell nuclei were stained with Hoechst 33342 (concentration) for 10 min. The tumors were finally rinsed in PBS and were mounted in an Antifade Mounting Medium (Vector laboratories, H-1000, Burlingame, CA). For imaging, excitation wavelength was set at 405 nm and fluorescent emission was recorded after filtration using a 415-450 nm band-pass filter. Several images were recorded along the z-axis for focal sectioning.

1.10. *In vivo* experiments

Animal experimentations were performed in accordance with European recommendations (Directive 2010/63/UE, September 22nd, 2010) and French regulations (Décret 2013-118, February 1st, 2013). They received the approval n°00465.02 from the French Ministry of Higher Education and Research in date of March 11th, 2014. Six to twelve weeks-old NMRI-nu (Rj:NMRI-Foxn1nu/Foxn1nu) or Balb/cJRj female mice were purchased from Janvier Labs (Le Genest Saint Isle, France). Behavior and weight of mice were monitored through out the experiment. Paracetamol (Doliprane, Sanofi-Aventis, Paris) was given at a dose of 1 mg/ml in the drinking water until the end of the experimentation. Injections were always performed under 1 to 3% isoflurane anesthesia (Axience Laboratories, Pantin, France). At the end of experiments, euthanasia was obtained by cervical dislocation under general anesthesia.

1.11. Cytokine and Hepatic enzymes analysis

Balb/cJRj mice (20 g) were randomized in siRNA-(7 mice) and polyplex-treated (8 mice) groups. They received a single 100 µL intravenous injection in the lateral tail vein of the control siRNA alone (siC: 1 mg/kg mouse body weight) or siC/πPEI polyplex N/P 14 (1 mg

siC, 3.5 mg πPEI/kg mouse body weight) (n=8). Blood samples were harvested on dry capillary tubes by retro-orbital puncture under general isoflurane gaz anesthesia 3h, 6h, 24h and 48h after injection. The plasma levels of hepatic enzymes (LDH, ASAT, ALAT) and some cytokines (IL-1β, IL-6, IL-12, TNF-α and IFN-γ) were determined using an external service facility (Mouse Clinical Institute, IGBMC, Illkirch France).

1.12. Subcutaneous hepatocellular carcinoma model

The subcutaneous and orthotopic cell-Derived Xenograft HCC mouse models have been previously characterized and reported.[3] The Huh-7 cell line (Japanese Collection of Research Bioresources Cell Bank, Osaka, Japan) was stably transformed to express a firefly luciferase by transduction of firefly luciferase-encoding pCLNCX vector (Dr. Lorang, NIH, Bethesda, MD, USA) with a retrovirus. Important to mention, this firefly luciferase was not silenced by SiC. These Huh-7luc cells (10^6 in 50 µL PBS) were subcutaneously injected on the back of NMRI-nu mice. Three weeks later, when the subcutaneous tumors reached at least 5 mm in one dimension, mice were anesthetized by isoflurane gaz inhalation and they were intraperitoneally injected with a solution of D-luciferin (Caliper Lifesciences) (2 mg in 100 µL PBS). The bioluminescence was monitored using an IVIS 50 *in vivo* imaging system (Caliper Lifesciences, Roissy, France) and, after bioluminescence acquisition, mice were allocated to the different experimental or control groups as described [4]. The polyplexes N/P 14 (20 µg siRNA in 50 µL 4.5% glucose solution) were injected into the tumors of still anesthetized mice. The mice were then let to awake. Longitudinal luminescence monitoring and intratumoral injections were repeated thrice a week for two weeks. Luminescence was calculated using the Living Image 3.1 software (Caliper Lifesciences) and was expressed as photons.second^{-1}.cm^{-2}.steradian^{-1} (p.s^{-1}cm^{-2}.sr^{-1}). The relative tumor growth (RTG) was calculated by dividing the luminescence at each time point to the initial value. Results were

expressed as mean ± standard error (SE). One-way analysis of variance (ANOVA) was completed with a post hoc multiple comparison analysis between groups using the Mann-Whitney or Fisher tests. Differences were considered significant at $p < 0.05$.

Alternatively, three weeks after intra-hepatic Huh-7-Luc cell implantation (10^6 in 50 µL PBS), PBS or 20 or 40 µg fluorescent Cy5-siRNA/πPEI were intravenously injected. 24h later, mice were injected with luciferin, livers were harvested and separated from the hepatic tumors. The tumor bioluminescence and the fluorescence of the siRNA were then recorded.

1.13. Histology

Tumors were harvested from euthanized mice and were fixed in 10% buffered formalin. Tissues were then embedded in paraffin, sectioned in 7 µm slices and stained with Hematoxylin and Eosin (H&E). The necrosis level was blindly scored from 0 (no necrosis) to 5 (complete necrosis).

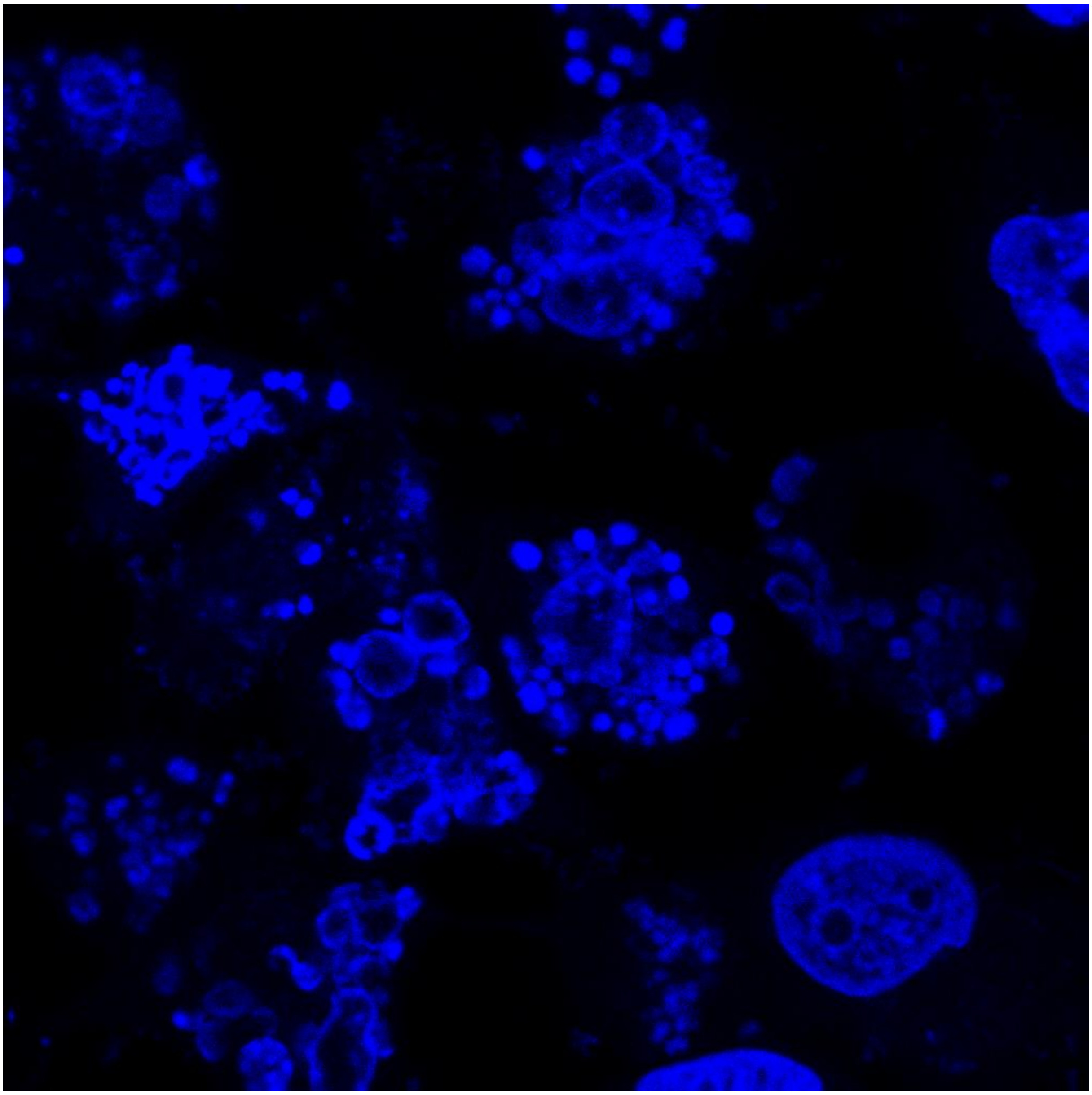

Figure S1. Cellular response to siRNA-mediated PLK1 mRNA degradation 3 days after addition of siRNA/πPEI onto Huh-7 2D culture. The morphology of the cell nuclei was observed after staining with Hoechst 33342 and fixation with 2.5% glutaraldehyde. Final concentrations were 100 µM πPEI and 20 nM siRNA and the polyplexes were added by simple dilution into the complete 10% FBS cell culture medium. Images showed nucleus fragmentation signaling apoptosis.

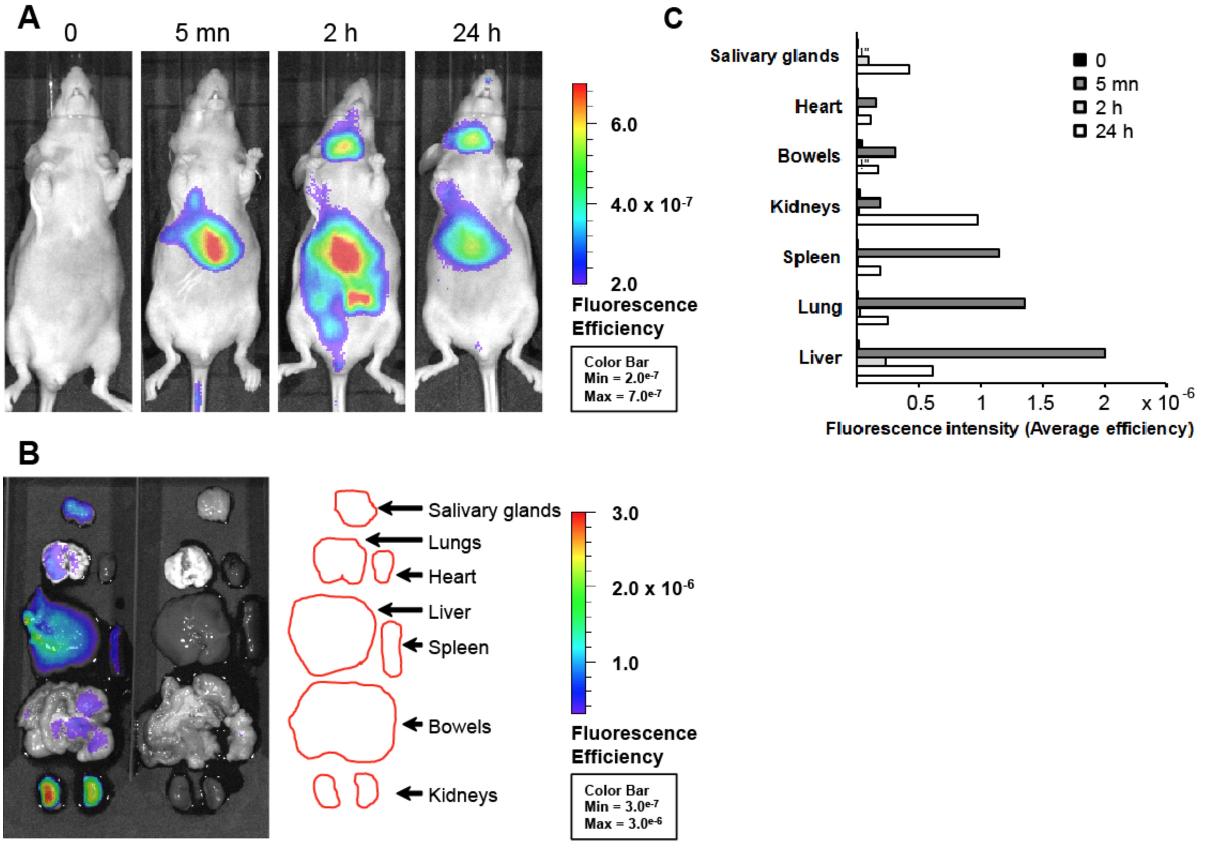

Figure S2. A. Kinetic of biodistribution of pPEI after i.v. injection of 2 mg Cy5-siRNA/pPEI in one representative mouse. B. Fluorescence of organs harvested 24h after injection of 2 mg Cy5-siRNA/pPEI (left) or PBS (right). C. Fluorescence quantification in different organs harvested at the indicated time points after i.v. injection (one mouse/time point). *: not tested (salivary glands, 5 mn; bowels, 2h)

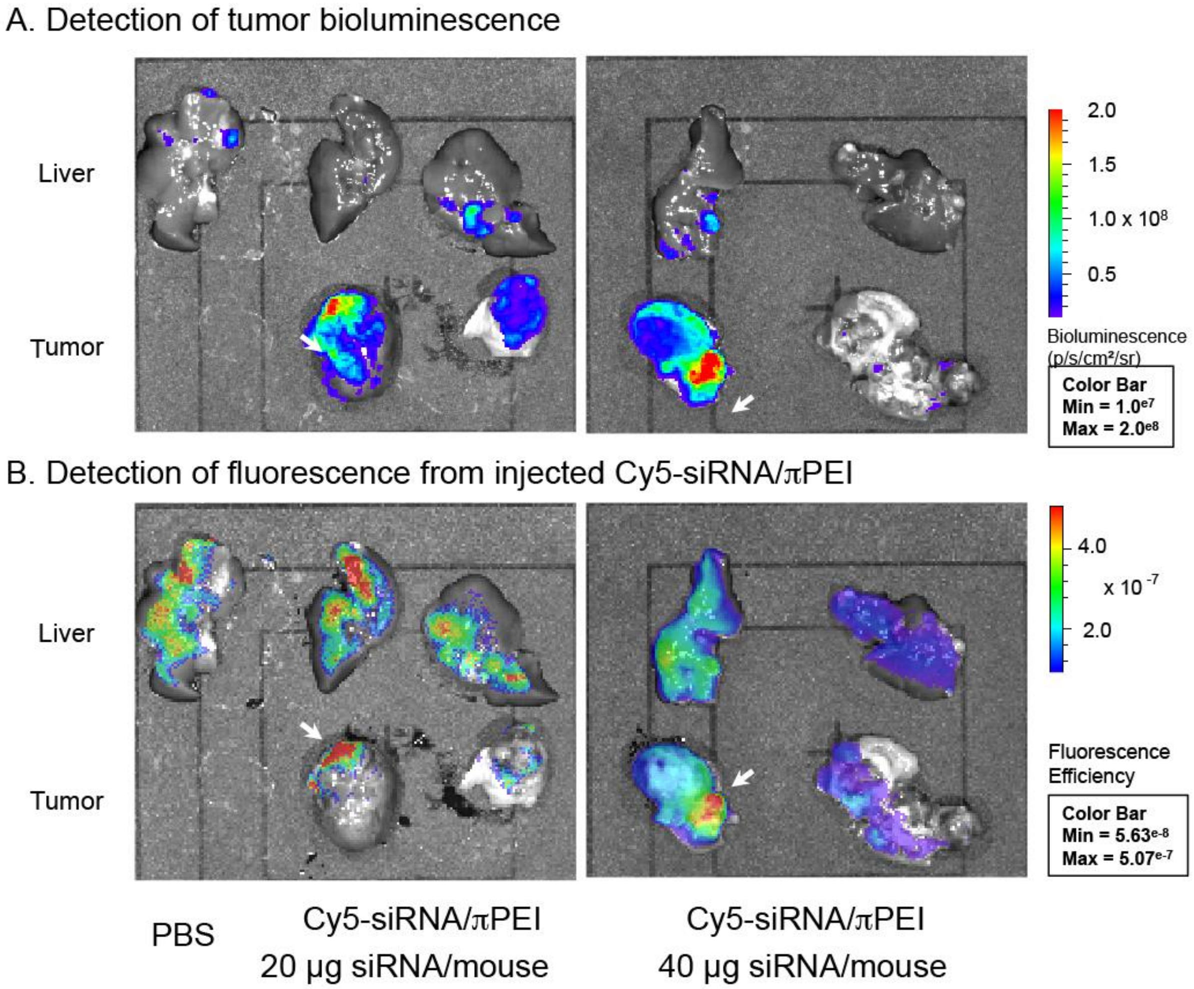

Figure S3. Distribution of Cy5-siRNA/πPEI between liver and Huh-7-Luc orthotopic tumor. NMRI-Nude mice with orthotopic Huh-7-Luc tumor were intravenously injected with PBS or the Cy5-siRNA/πPEI (N/P 14) at doses of 20 µg and 40 µg siRNA/mouse. 24h later, mice were injected with luciferin, livers were harvested and separated from the hepatic tumors. The tumor bioluminescence (upper panels) and then the fluorescence of the siRNA (lower panels) were recorded. Fluorescence is seen in the liver and in the tumors with clear colocalization with bioluminescence (arrows) in part of tumor.

A

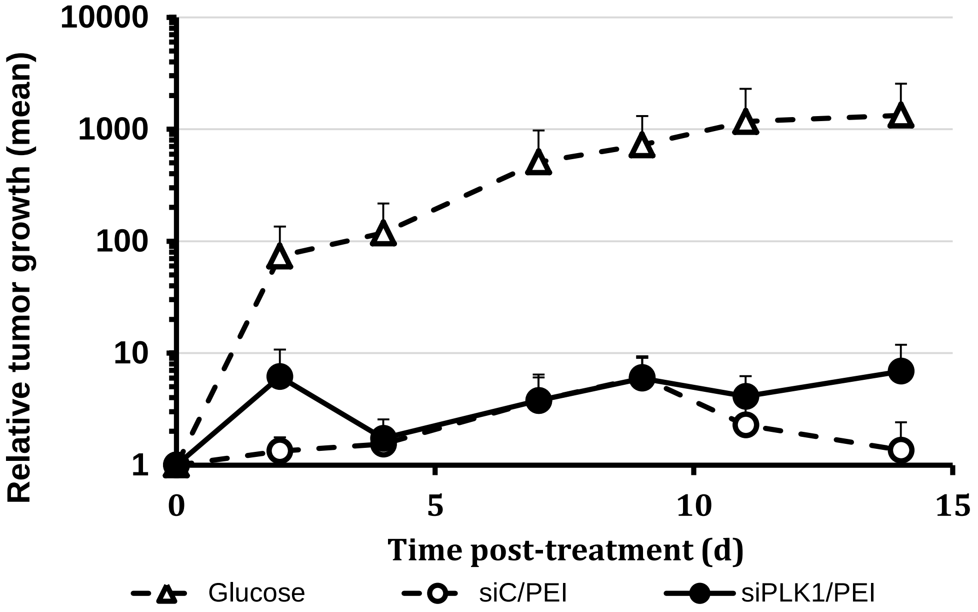

B

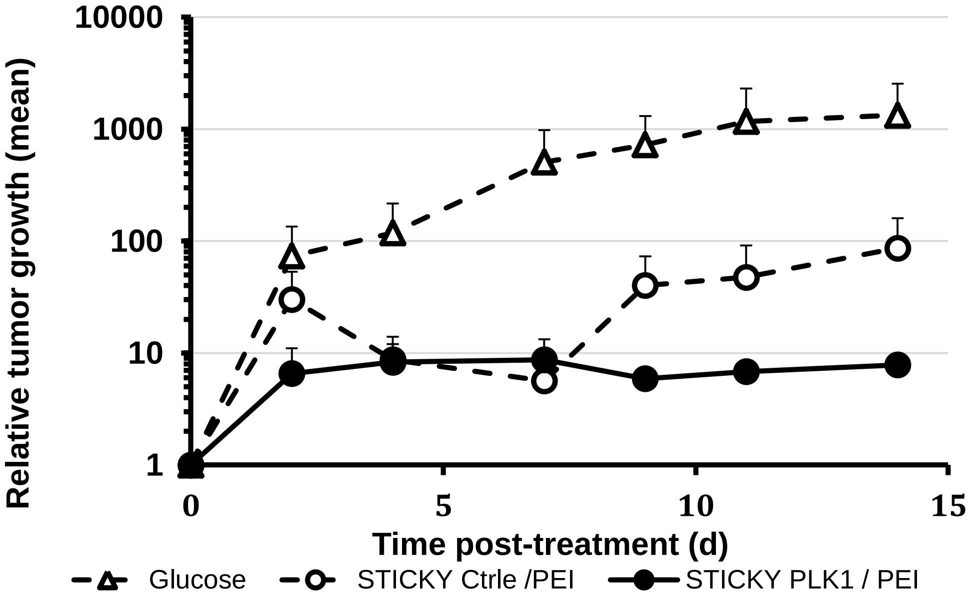

Figure S4. Quantification of relative tumor growth following administration of polyplexes in a mice model of hepatocellular cancer. The indicated polyplexes containing 20 µg nucleic acids were repeatedly injected into tumors over two weeks at days 0, 2, 4, 7, 9 and 11. A. Histograms A and B report the relative tumor growth following administration of siRNA/PEI polyplexes (N/P10) or sticky siRNA/PEI polyplexes, respectively. The sticky siRNA is supposed to facilitate siRNA delivery[5]

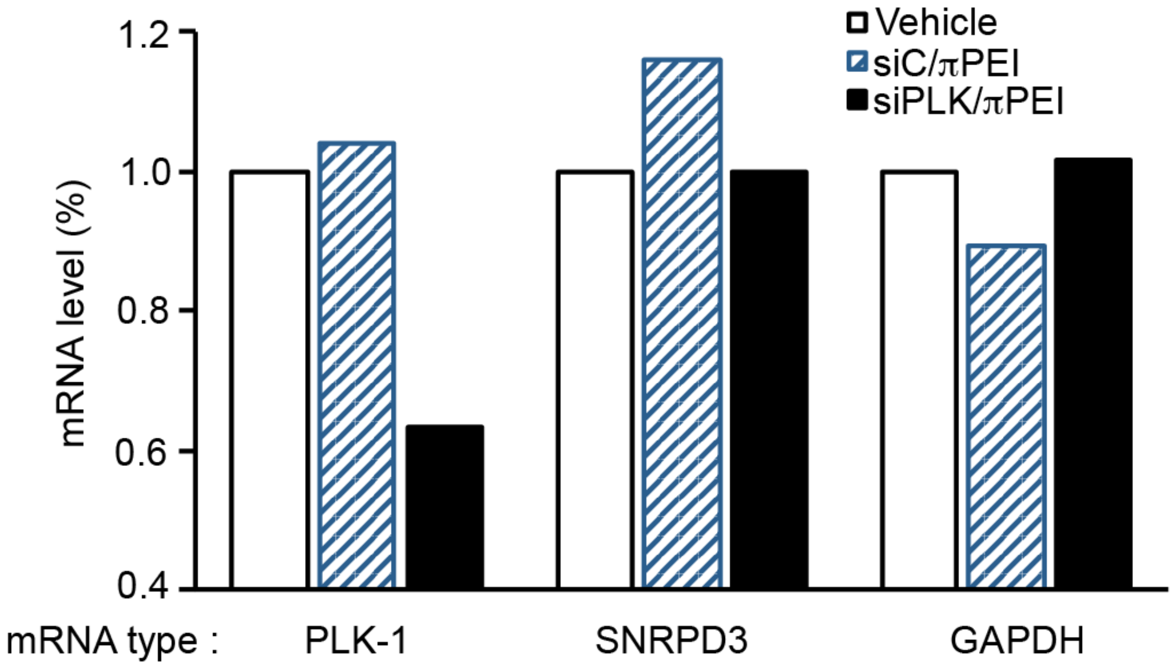

Figure S5. Quantification of various selected mRNA levels in tumors after intratumoral administration of vehicle (white bars), siC/πPEI (hatched blue bars) and siPLK/πPEI polyplexes (black bars). The SNRPD3 and GAPDH are sentinel genes and were used for normalization. The mRNA levels were determined by quantitative RT-PCR.

References

[1] G. Creusat, J. S. Thomann, A. Maglott, B. Pons, M. Dontenwill, E. Guerin, B. Frisch, G. Zuber, *J. Control. Release* **2012**, *157*, 418-426.

[2] A. D. Judge, M. Robbins, I. Tavakoli, J. Levi, L. Hu, A. Fronda, E. Ambegia, K. McClintock, I. MacLachlan, *J. Clin. Invest.* **2009**, *119*, 661-673.

[3] T. Wu, E. Heuillard, V. Lindner, G. Bou About, M. Ignat, J. P. Dillenseger, N. Anton, E. Dalimier, F. Gosse, G. Foure, F. Blindauer, C. Giraudeau, H. El-Saghire, M. Bouhadjar, C. Calligaro, T. Sorg, P. Choquet, T. Vandamme, C. Ferrand, J. Marescaux, T. F. Baumert, M. Diana, P. Pessaux, E. Robinet, *Sci. Rep.* **2016**, *6*, 35230.

[4] C. Leboeuf, L. Mailly, T. Wu, G. Bour, S. Durand, N. Brignon, C. Ferrand, C. Borg, P. Tiberghien, R. Thimme, P. Pessaux, J. Marescaux, T. F. Baumert, E. Robinet, *Mol. Ther.* **2014**, *22*, 634-644.

[5] A. L. Bolcato-Bellemin, M. E. Bonnet, G. Creusat, P. Erbacher, J. P. Behr, *Proc. Natl. Acad. Sci. U. S. A.* **2007**, *104*, 16050-16055.
